# Supplementary material for: From the first touch to biofilm establishment by the human pathogen Candida glabrata: a genome-wide to nanoscale view
Source: Commun Biol. 2021 Jul 20;4:886. doi: 10.1038/s42003-021-02412-7 (PMC8292413; doi:10.1038/s42003-021-02412-7)
Supplement: Supplementary file 2 — Supplementary Information [file 42003_2021_2412_MOESM2_ESM.pdf]

Supplementary Information

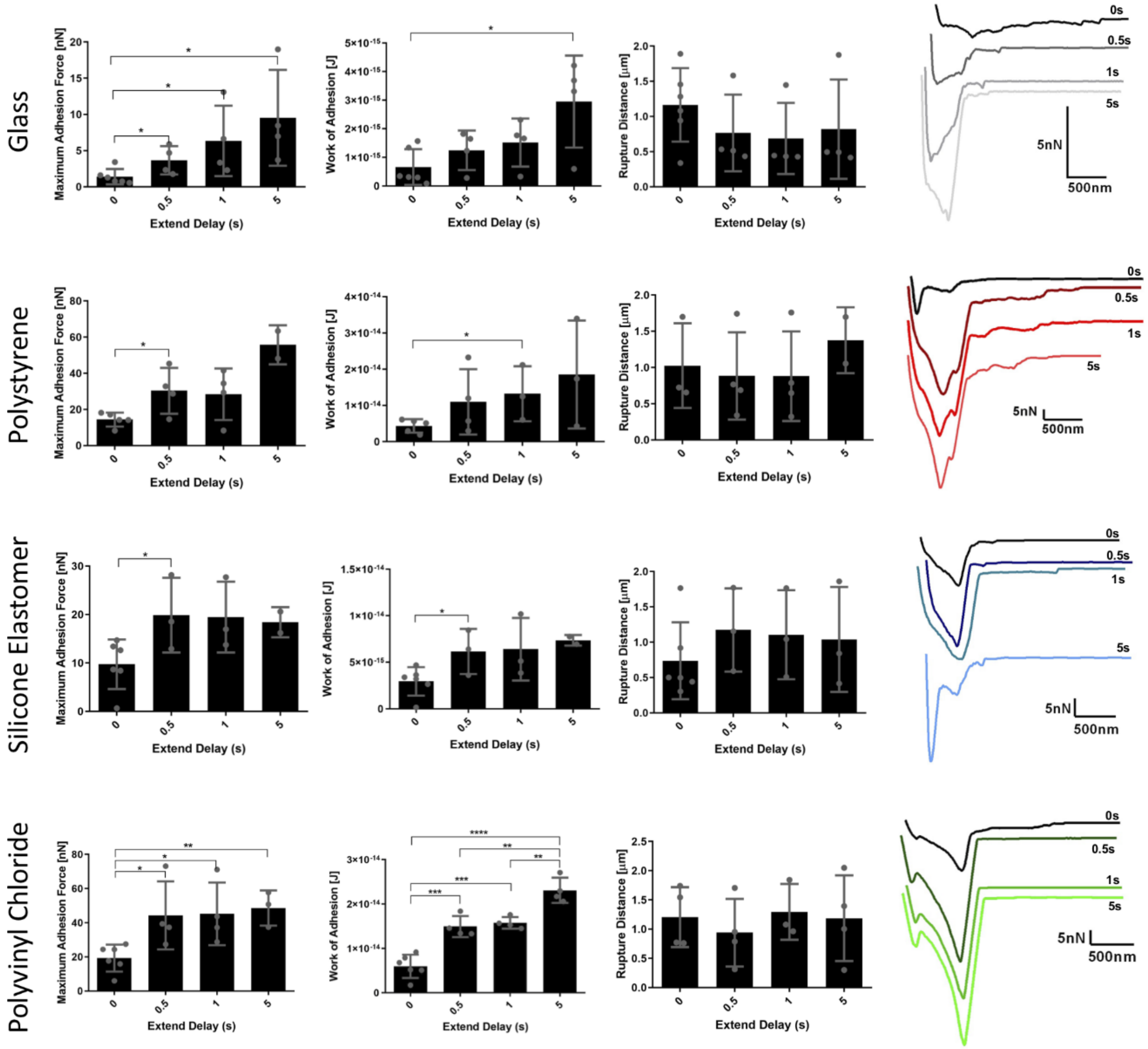

Supplementary Fig. 1 Interaction of *C. glabrata* wild-type strain KUE100 with glass, polystyrene, silicone elastomer and polyvinyl chloride by SCFS, using different contact times: 0s, 0.5s, 1s and 5s. Characterization of these interactions is based on the maximal adhesion force, work of adhesion and rupture distance, with the representative force-distance curves for each material and for each contact time (the lighter the force curve, the higher the contact time). Horizontal lines indicate the average levels from at least 3 yeast cells, from at least 2 independent cell cultures, immobilized on the cantilever for the interaction with each material. 256, 100 and 25 force-distance curves were recorded for the interaction with the materials, with 0s, 0.5-1s and 5s, respectively. Error bars indicate standard deviations. *, $P<0.05$; **, $P<0.01$; ***, $P<0.001$; ****, $P<0.0001$, $n \geq 3$, except for the level of rupture distance and maximum adhesion force on polystyrene 5s extend delay, and maximum adhesion force and work of adhesion on silicone elastomer 5s extend delay, which were not considered for statistical analysis.

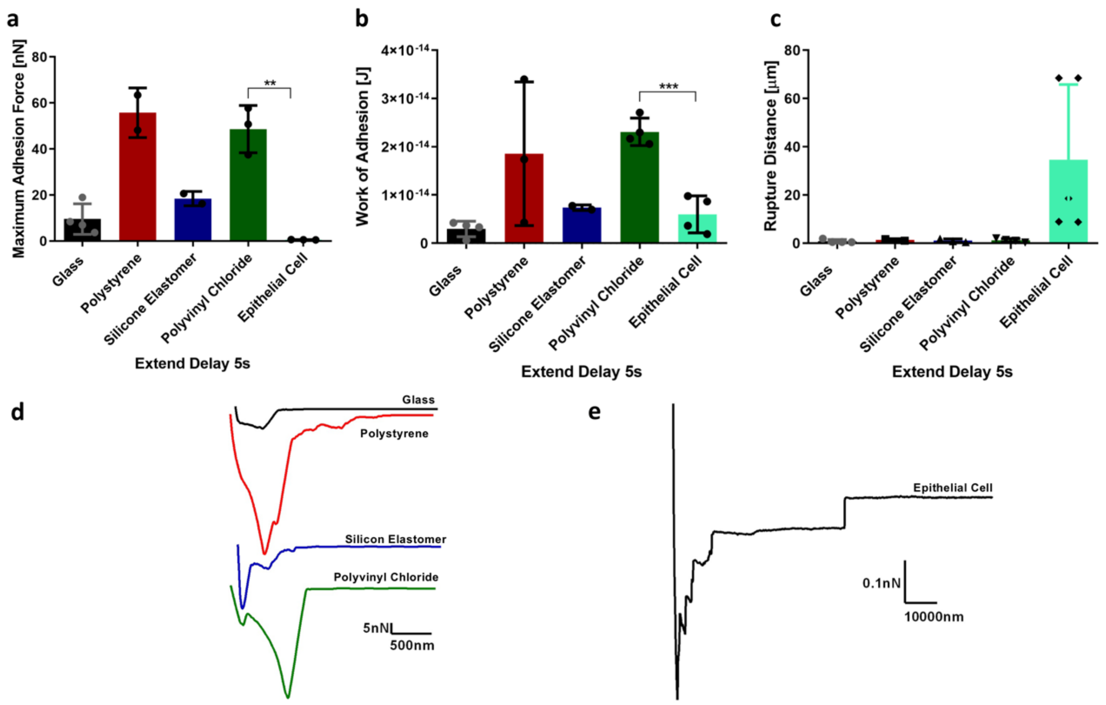

Supplementary Fig. 2 Comparison of *C. glabrata* wild-type KUE100 strain interaction with surface materials and human vaginal epithelial VK2/E6E7 cells, with 5s of contact time. Average of the **a** maximal adhesion force, **b** work of adhesion and **c** rupture distance measured on each retraction curve. **d** Representative force-distance curves of the interaction with glass (black), polystyrene (red), silicone elastomer (blue) and polyvinyl chloride (green). **e** Representative force-distance curve of the interaction with epithelial cells. For every condition, at least 4 yeast cells, from at least 3 independent cell cultures, were immobilized on the cantilever for the interaction. Error bars indicate standard deviations. **, $P<0.01$; ***, $P<0.001$, $n \geq 3$, except for the level of polystyrene **a** and **c,** and silicone elastomer **a** and **b,** which were not considered for the statistical analysis.

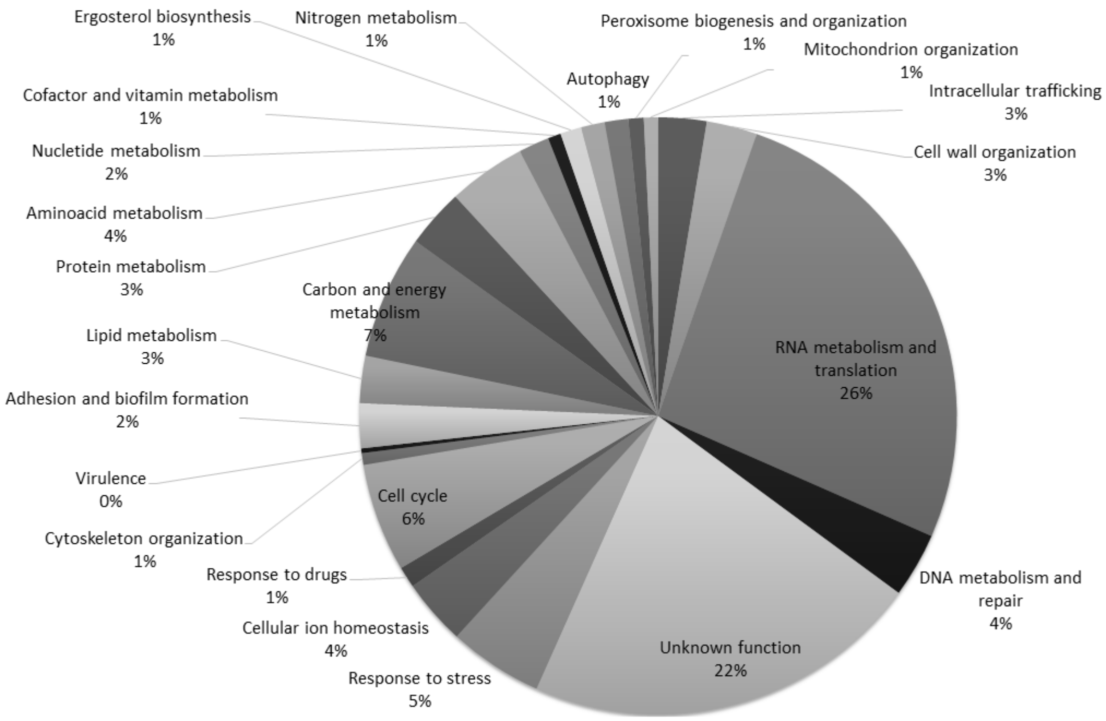

Supplementary Fig. 3 Categorization and frequency of the genes up-regulated in *C. glabrata* cells upon 24h of biofilm growth, given by the KUE100 wild-type cells grown in biofilm in relation to KUE100 wild-type cells grown in planktonic conditions, based on the biological process taxonomy of gene ontology (p-value<0.05).

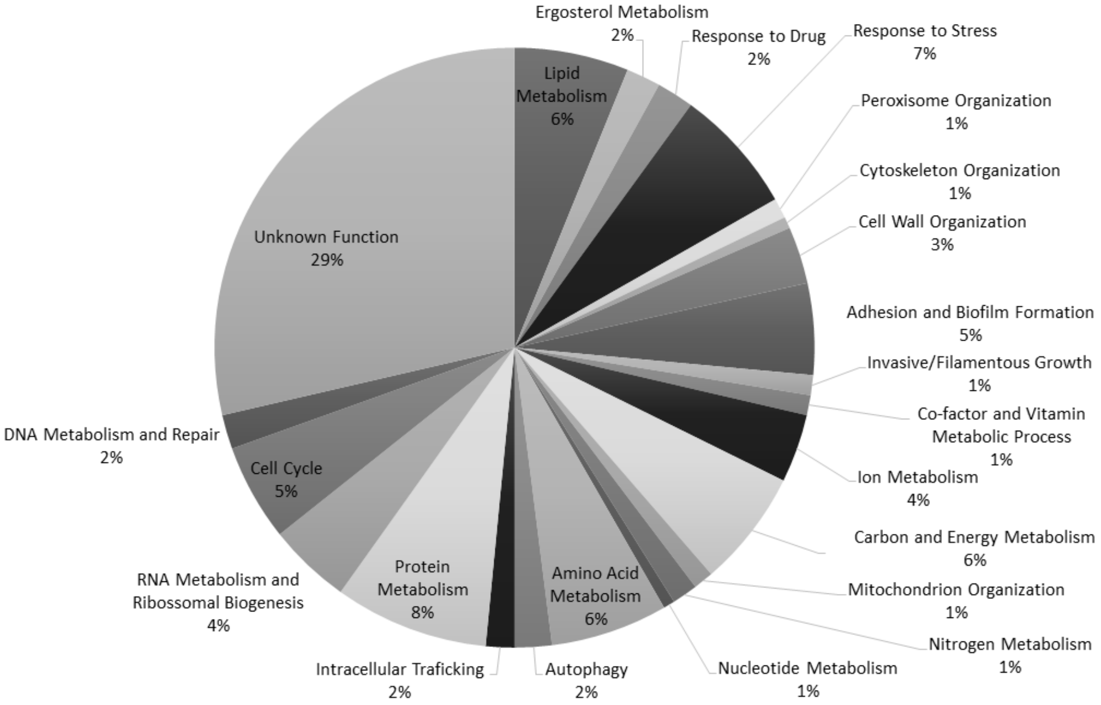

Supplementary Fig. 4 Categorization and frequency of the genes up-regulated by CgEfg1 upon biofilm growth in *C. glabrata*, given by the *Δcgefg1* deletion mutant cells in relation to KUE100 wild-type cells grown in biofilm, based on the biological process taxonomy of gene ontology (p-value<0.05).

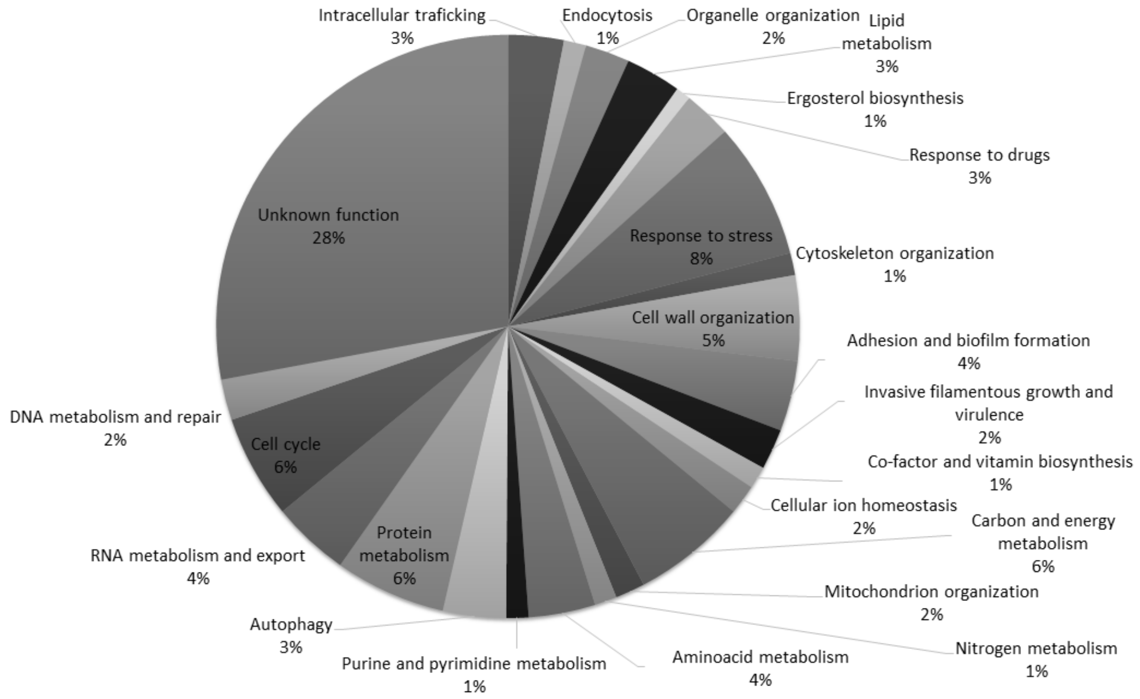

Supplementary Fig. 5 Categorization and frequency of the genes up-regulated by CgTec1 upon biofilm growth in *C. glabrata*, given by the *Δcgtec1* deletion mutant cells in relation to KUE100 wild-type cells grown in biofilm, based on the biological process taxonomy of gene ontology (p-value<0.05).

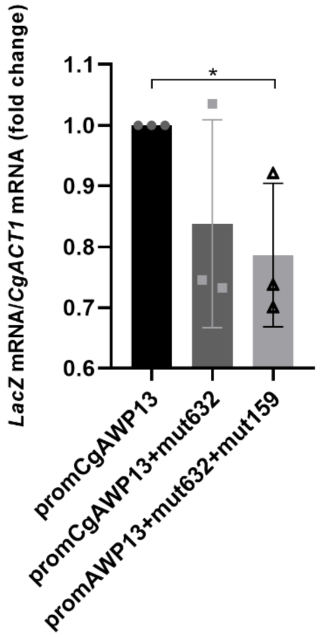

Supplementary Fig. 6 Comparison of the variation of lacZ transcript levels determined by RT-PCR in L5U1 cells harboring the pYEP354_promCgAWP13_lacZ, the pYEP354_promCgAWP13+mut632_lacZ or the pYEP354_promCgAWP13+mut632+mut159_lacZ plasmids in biofilm conditions. Transcript levels *CgACT1* were used for normalization. Expression values are the average of at least three independent experiments. Error bars represent the corresponding standard deviations. *, P<0.05, n ≥ 3.

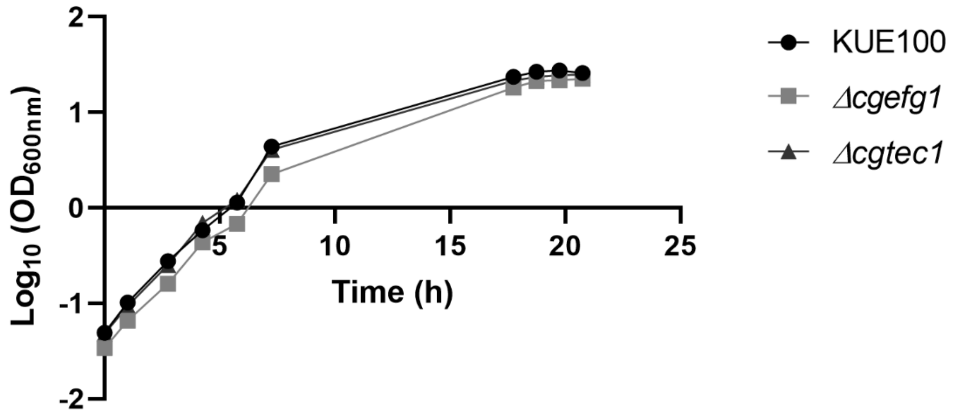

Supplementary Fig. 7 Growth curves of the *C. glabrata* wild-type strain and derived deletion mutants Δcgefg1 and *Δcgtec1*, at SDB medium, pH 5.6, measured in terms of variation of Optical Density at 600nm.

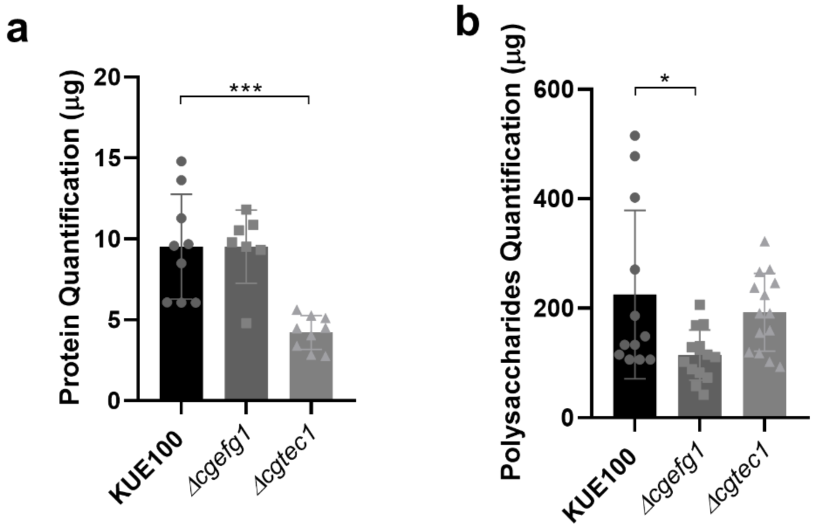

Supplementary Fig. 8 Quantification of **a** proteins and **b** polysaccharides from the ECM of *C. glabrata* wild-type and derived deletion mutants Δ*cgefg1* and *Δcgtec1* biofilms grown for 48h in SDB medium, pH 5.6. The values obtained are the average of at least three independent experiments. Error bars represent the corresponding standard deviations. *, $P<0.05$; ***, $P<0.001$, $n \geq 7$.

Supplementary Table 1 Adhesin-encoding genes activated upon *C. glabrata* KUE100 biofilm formation versus planktonic growth, repressed upon the absence of *CgEFG1* and *CgTEC1* genes upon *C. glabrata* planktonic growth and biofilm formation.

**Gene Name**	**Description**	**MW (kDa)**	**Log2 Fold Change wild-type: biofilm vs planktonic**	**Log2 Fold Change Planktonic: wild-type vs Δ*cgefg1***	**Log2 Fold Change Planktonic: wild-type vs Δ*cgtec1***	**Log2 Fold Change Biofilm: wild-type vs Δ*cgefg1***	**Log2 Fold Change Biofilm: wild-type vs Δ*cgtec1***
PWP5	Cell wall adhesin; predicted GPI anchor; contains tandem repeats; belongs to adhesin cluster II	108.8	5.92			-4.95	-2.44
AED2	Putative adhesin; predicted GPI-anchor; belongs to adhesin cluster III	104.2	4.66			-3.19	-2.57
AWP13	Predicted cell wall adhesin with a role in adhesion; belongs to adhesin cluster III; predicted GPI anchor; contains tandem repeats	138.9	4.41			-3.5	-2.33
PWP1	Protein with 32 tandem repeats; putative adhesin-like protein; belongs to adhesin cluster II	473.6	3.8			-2.23	
EPA20	Putative adhesin; belongs to adhesin cluster I	98.3	3.18			-1.55	
PWP3	Protein with tandem repeats; putative adhesin-like protein; belongs to adhesin cluster II	148.9	2.94			-1.99	
EPA3	Epithelial adhesion protein; belongs to adhesin cluster I; GPI-anchored	99.6	2.91				
AWP1	Adhesin-like protein; identified in cell wall extracts by mass spectrometry; belongs to adhesin cluster VI; predicted GPI anchor	84.3	2.91	-2.83		-2.17	
EPA2	Epithelial adhesion protein; predicted GPI-anchor; belongs to adhesin cluster I	144.3	2.43				
AWP6	Adhesin-like protein; identified in cell wall extracts by mass spectrometry; belongs to adhesin cluster IV; predicted GPI anchor	58	2.32				
EPA10	Putative adhesin-like protein; belongs to adhesin cluster I	148.1	1.89			-1.25	-1.42
AWP3	Putative adhesin-like protein; identified in cell wall extracts by mass spectrometry; belongs to adhesin cluster VI	143.6	1.82			-1.45	
EPA12	Putative adhesin-like cell wall protein; belongs to adhesin cluster I	99.5	1.76			-1.49	
EPA1	Sub-telomerically encoded adhesin with a role in cell adhesion; GPI-anchored cell wall protein; N-terminal ligand binding domain binds to ligands containing a terminal galactose residue; belongs to adhesin cluster I	111.7	1.72				
AED1	Adhesin-like protein required for adherence to endothelial cells; identified in cell wall extracts by mass spectrometry; belongs to adhesin cluster III; predicted GPI anchor; 6 tandem repeats; expressed more in stationary growth phase	112.6	1.71			-2.1	-1.38
EPA9	Putative adhesin; belongs to adhesin cluster I	154.7	1.66			-1.05	-1.45
AWP4	Putative adhesin-like protein with internal repeats; identified in cell wall extracts by mass spectrometry; ORF appears artificially broken into fragments due to sequencing errors; belongs to adhesin cluster V; predicted GPI anchor	-	1.23	-1.59		-1.98	
EPA23	Predicted GPI-linked adhesin-like protein; belongs to adhesin cluster I	94.5	1.07				
PWP2	Adhesin-like protein with tandem repeats; contains a PA14 domain; belongs to adhesin cluster II	135.2	1.06				
EPA11	Putative adhesin; belongs to adhesin cluster I	176.9	-4.19	-2.79	-1.36		
EPA13	Sub-telomerically encoded lectin-like adhesin with a role in cell adhesion; contains multiple tandem repeats; predicted GPI-anchor; belongs to adhesin cluster I	111.9	-4.92	-1.92	-1.17		
EPA8	Putative adhesin-like protein; belongs to adhesin cluster I	82.8		-1.54			
EPA6	Sub-telomerically encoded adhesin with a role in cell adhesion; binds to ligands containing a terminal galactose residue; expressed during murine urinary tract infection, biofilm-upregulated; belongs to adhesin cluster I	77.8		-1.34			
EPA7	Adhesin-like protein with a predicted GPI-anchor; belongs to adhesin cluster I; strain BG14 encodes the full-length protein, whereas CBS138 contains a stop codon that terminates the ORF at codon 379	77.9		-1.17			

Supplementary Table 2. List of primers used in this study.

**Name**	**Sequence (5'-3')**
***CgEFG1* gene cloning**	
pGREG_CgEFG1_Fw	GAATTCGATATCAAGCTTATCGATACCGTCGACAATGTCTGAAAGAGAATTGCCTG
pGREG_CgEFG1_Rv	GCGTGACATAACTAATTACATGACTCGAGGTCGACTTACATGTGGTGATGTATTTGG
***CgTEC1* gene cloning**	
pGREG_CgTEC1_Fw	GAATTCGATATCAAGCTTATCGATACCGTCGACAATGACTGTCTCAAATGACAGCT
pGREG_CgTEC1_Rv	GCGTGACATAACTAATTACATGACTCGAGGTCGACTCAGTTGGACTGAATACCTTGC
pGREG576 GAL1-to-PDCI promoter replacement	
pGREG_PDC1_Fw	TTAACCCTCACTAAAGGGAACAAAAGCTGGAGCTAGCATTTTTATACACGTTTTAC
pGREG_PDC1_Rv	GAAAAGTTCTTCTCCTTTACTCATACTAGTGCGGCTGTTAATGTTTTTTGGCAATTG
pGREG576 GAL1-to-MT-I promoter replacement	
pGREG_MT-I_Fw	TTAACCCTCACTAAAGGGAACAAAAGCTGGAGCTCTGTACGACACGCATCATGTGGCAATC
pGREG_MT-I_Rv	GAAAAGTTCTTCTCCTTTACTCATACTAGTGCGGCTGTGTTTGTTTTTGTATGTGTTTGTTG
**Deletion of *CgEFG1, CgTEC1***	
ΔCgEFG1_Fw	GGAGCAGGGAGTTACTGGTTAATGAGCGTAGACTTGAACTGAAAAGAAAATGTGCGGGCCGCTGATCACG
ΔCgEFG1_Rv	AACAATTCATTATGTTATACAATGGTACATAGCGATTCATTACGAATATTAAGTTACATCGTGAGGCTGG
ΔCgTEC1_Fw	AAGAGTACTAATACACATCGTACTCCCCCCCACAAATAACGCCCTCAATCTATATTGGCCGCTGATCACG
ΔCgTEC1_Rv	TCAGCAAAACATTTCTGCAGAAAAAATAAAAATGTAGCATTCCTACATCTCTCTCACATCGTGAGGCTGG
ΔCgEFG1_Fw_conf	GCCTGGATACACATACTTAC
ΔCgEFG1_Rv_conf	AACAGTAACTCCGTTGTG
ΔCgTEC1_Fw_conf	GACAGCTCGGTATCAGATAGGT
ΔCgTEC1_Rv_conf	GTGGAGATGATGCTTTCGAAGA
**RT-PCR experiments**	
CgACT1_Fw	AGAGCCGTCTTCCCTTCCAT
CgACT1_Rv	TTGACCCATACCGACCATGA
CgAWP13_Fw	TTAATATCTTGCTGGGCTTTTGG
CgAWP13_Rv	AGCGTAGCACTGTCTATGATTATTTCTT
CgPWP5_Fw	GGCTGGCTTTCGTGCAATA
CgPWP5_Rv	CGACGGACCCTTGTAAGATTGT
CgAED2_Fw	AAAGCCTCAATGGTATGACAGAAGAC
CgAED2_Rv	CAGATGAATTTTGGAATGGGAAA
